# The Association between Traditional Chinese Dietary and Herbal Therapies and Uterine Involution in Postpartum Women

**DOI:** 10.1155/2011/918291

**Published:** 2011-04-12

**Authors:** Ming Ho, Tsai-Chung Li, Shan-Yu Su

**Affiliations:** ^1^Department of Obstetrics and Gynecology, China Medical University Hospital, Taichung 40447, Taiwan; ^2^Biostatistics Center & Graduate Institute of Biostatistics and China Medical Science, China Medical University, Taichung 40402, Taiwan; ^3^Department of Chinese Medicine, China Medical University Hospital, Taichung 40447, Taiwan

## Abstract

*Background*. Traditional Chinese postpartum care is believed to help in the recovery of women after delivery. *Objective*. This study investigated the association of elements in dietary and herbal therapy with uterine involution. *Methods*. Indices of uterine involution were measured ultrasonographically in 127 postpartum women between 4-6 weeks after delivery. A self-reported retrospective questionnaire was used to query women about their frequencies of taking herbal medicines and consuming special diets during the first month after delivery. Correlation coefficients were calculated to identify the associations, then the regression models were used to identify the predictors. *Result*. Among the herbal medicines and diet, consumption of *Eucommia ulmoides *(*E. ulmoides*) negatively correlated with the AP diameter of the uterus and the cavity. *E. ulmoides* was also the only predictor of maximum AP diameter of the uterus, AP diameter of the uterus 5 cm from the fundus, and the maximum AP diameter of the cavity. Moreover, consumption of Sheng-hau-tang was significantly correlated with anteverted uterus and was a predictor of anteverted uterus. *Conclusion*. *E. ulmoides* and Sheng-hau-tang positively correlated with the degree of uterine involution after delivery, implying that both therapies might possess the pharmacological efficacy of uterine contraction in postpartum women.

## 1. Introduction

Uterine involution normally occurs within 6–8 weeks after delivery [1], although the speed and degree of uterine involution varies greatly from woman to woman [2]. Poor uterine involution has been shown to be closely related to maternal morbidity and, in particular, postpartum hemorrhage [3, 4]. Obstetric methods used to assist uterine involution include removal of the uterine contents by curettage and administration of uterotonic agents [5]. A method used by the Chinese to help in the recovery of the uterus is to practice traditional Chinese postpartum care [6], which is also called doing-the-month, a custom that originated in China but is practiced in several areas of Asia and in Western countries with Chinese migrants [7–9].

Traditional Chinese postpartum care is comprised of three dimensions: behavior therapy, dietary therapy, and herbal therapy [10]. According to the dietary and herbal therapy, during the first month after delivery, the consumption of food considered to be functionally “hot,” “cold,” and “sour” in nature is also restricted [11, 12]. Base on Chinese medical theory, “hot” foods induce irritation, sweating, and bleeding, “cold” foods induce a cold feeling and decreased organ functions, and “sour” foods induce astringency and interfere with the expulsion of lochia. These extreme foods are considered potentially harmful to postpartum women. In contrast, food considered to be “warm” can nourish organs [13]. Therefore, postpartum women are encouraged to consume traditional “warm” dishes, including chicken soup and sesame oil chicken, as well as “warm” herbal medicines [10, 14, 15]. During the first postpartum month, mothers consume a range of herbs, including Sheng-hau-tang, Si-wu-tang, and *Eucommia ulmoides (E. ulmoides)*. The warm food and herbal medicines are believed to promote wound healing, prevent infection, chronic illness, and increase the quality and quantity of milk for breast feeding [16–18].

Although traditional Chinese postpartum care has been practiced for hundreds of years, evidence-based literature on Chinese herbs and foods pertaining to postpartum care is very limited. Most of the studies were done by sociologists and anthropologists and merely described the items, the methods, and the beliefs of Chinese customs [14, 19, 20]. The only epidemiological study conducted on traditional Chinese postpartum care found that engaging in the doing-the-month practice is associated with fewer physical symptoms and lower odds of postpartum depression [11]. All of those studies regarded the traditional Chinese postpartum care as one integrated activity and reported that woman should be engaged in all aspects to gain benefits from the customs. We hypothesized that the effects of different dimensions and elements on uterine involution are not the same. 

In this study, we aimed to evaluate the impact of foods and herbs of traditional Chinese postpartum care on uterine involution. We evaluated three types of food nature, two traditional dishes, and three herbal medicines separately in order to find out the most important factor that might help in the recovery of the uterus. Correlation analysis between the use of each herb and diet and the indices of uterine involution was performed. The uterine involution indices were recorded 4–6 weeks after delivery, and the frequencies of each diet and herb during the first month after delivery were assessed by a retrospective questionnaire.

## 2. Materials and Methods

### 2.1. Subjects

Study subjects comprised women undergoing a 4–6 week postpartum followup examination at the obstetric outpatient clinic of the China Medical University Hospital (CMUH; Taichung, Taiwan) between February 2008 and January 2009. All women received a full explanation of the study and provided written informed consent. Inclusion criteria included receipt of ante partum care at the CMUH, age ≥ 20 years, gestational age ≥ 36 weeks, and an infant birth weight ≥ 2500 g. Women were excluded if they were pregnant; had a history of chronic disease requiring medication, including hypertension, diabetes, and cancer; were smokers or drug addicts. This study was approved by the Institutional Review Board of the CMUH.

### 2.2. Measurement of Consumption of Foods and Herbal Medicines

The frequency of eating a specific food or taking a herbal medicine during the first 30 days after delivery was measured retrospectively by a self-reported questionnaire. The herbal and dietary variables included 3 categories and one summative index. The 3 categories were food nature, dish, and herb. Variables measured in the food category included iced or cold drinks, “cold” food, “hot” food, and “sour” food. Variables in the dish category included chicken and sesame oil chicken. Variables in the herb category included Sheng-hau-tang, Si-wu-tang, and *E. ulmoides*. All the variables are ordinarily measured. For each variable in the food nature category, no consumption in the first postpartum month was scored as 0. Total consumption for 1–3 days was scored as 1, for 4–7 days was scored as 2, and for more than 7 days was scored as 3. A list of hot food (deep fried foods, pepper, chili pepper, curry, pungent foods, and barbecue sauce), cold food (uncooked vegetable, grapefruit, watermelon, coconut, tangerine, tomato, mung beans, lotus root, cucumber, Chinese cabbage, daikon radish, and bean curd), and sour food (lemon, vinegar, plum, and sour fruit) were provided to the women. For chicken soup and sesame oil chicken, the consumption for 0–3 days was scored as 0, for 4–7 days was scored as 1, for 8–15 days was scored as 2, and consumption for more than 15 days was scored as 3. The sesame oil chicken is comprised of chicken, ginger, black sesame oil, and rice wine [21], and the chicken soup was defined as a soup cooked by boiling chicken until well cooked. No consumption of Sheng-hau-tang and Si-wu-tang was scored as 0. Consumption of Sheng-hau-tang and Si-wu-tang for 1–4 days was scored as 1, for 5–10 days was scored as 2, and for more than 10 days was scored as 3. The ingredients of Sheng-hau-tang and Si-wu-tang were listed in Table 1. No consumption of *E. ulmoides* during the first postpartum month was scored as 0. Consumption of *E. ulmoides* for 1–7 days as scored as 1, for 8–21 days was scored as 1, and for more than 21 days was scored as 3. Lastly, the total number of days that the women took Chinese medicines, including the above-mentioned medicines and any other Chinese medicines, was scaled as 0 if women did not consume any of them. The taking of Chinese medicine for 1–5 days was scored as 1, that for 5–15 days was scored as 2, and that for more than 15 days was scored as 3. The scales were designed by three Chinese Medical professors according to commonly used frequencies.

### 2.3. Measurement of Uterine Involution

**Figure 1:**
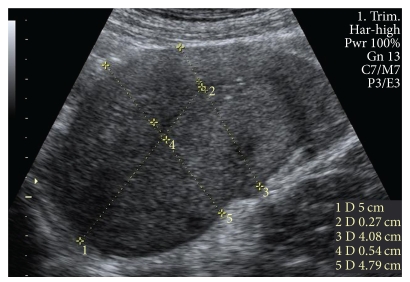
Representative ultrasonographic image illustrating how the AP diameter of the uterus and the cavity were measured. (1) measured 5 cm from the fundus; (2) AP diameter of the cavity 5 cm from the fundus; (3) AP diameter of the uterus 5 cm from the funus; (4) maximum AP diameter of the cavity; (5) maximum AP diameter of the uterus.

Uterine involution was measured ultrasonographically between day 28 and day 42 after delivery. Maximum anteroposterior (AP) diameter of the uterus, AP diameter of the uterus 5 cm from the fundus, maximum AP diameter of the cavity, and AP diameter of the cavity 5 cm from the fundus were measured in a longitudinal view (Figure 1). The presence of fluid, heterogeneous contents, of gas/hypoechogenic foci in the uterine cavity, as well as the presence of anteverted uterus were also measured and designated as 0 (no) and 1 (yes). All the ultrasound examinations were performed with commercially available real-time machines (Voluson 730 Pro, GE Medical system Kretztechnik GmbH & Co. OHG, Zipf, Austria) with 2–7 MHz transabdominal AB2-7, Curved Array Probe. The mother had a moderately filled bladder when examined. Tender compression by the probe was used, and the measurement was made.

### 2.4. Statistical Analysis

Statistical analyses were performed using the SPSS 13.0 statistical software package. Individual variables were examined by percentage, mean, and standard deviation (SD). Associations between the AP diameters of the uterus and cavity and each herbal variable, and between the same AP diameters and each dietary variable were conducted using Pearson's correlation, followed by a multivariate linear regression to determine significant predictors for the AP diameter of the uterus and the cavity. Associations between the presence gas/hypoechogenic foci and anteverted uterus and each herbal variable, and between the same indices and each dietary variable were tested using biserial correlation, followed by logistic regression to determine significant predictors for the presence of gas/hypoechogenic foci and anteverted uterus. Diet and herb variables of the same category were entered into the model at the same time. The entered method was applied in both multivariate linear regression and logistic regression model. The difference in AP diameter of uterine and cavity between women consuming different level of *E. ulmoides* were examined by analysis of variance (one-way ANOVA). A two-tailed *P*-value of <.05 was considered to be statistically significant in the regression model.

## 3. Results

### 3.1. Subjects

Among the 149 women who were asked to participate in the study, 2 refused to participate, and 20 did not finish all the questionnaires. Therefore, a total of 127 women participated in the study. The mean age of the 127 recruited women was 30.5 ± 3.9 years (range, 22–41 years). They had a mean parity of 1.4 ± 6.3 (range, 1–4) and gestational age of 38.3 ± 1.4 weeks (range, 36–41 weeks). The characteristics of the enrolled women, including the method of delivery, education level, employment status, living environment, and the place of herb purchase, are listed in Table 2. All women received oxytocin and methylergonovine maleate intravenously as a routine immediately after the delivery of the placenta. Extra prescribed uterotonic agents and antibiotics were also listed.

### 3.2. Diet and Herbal Use during the First Month after Delivery

There were 78% of our subjects who did not drink any iced or cold drinks during the first month after delivery. Avoiding eating “cold” foods and “hot” foods was also followed during this time period; however, the majority of women did not avoid “sour” foods (Table 3). The majority of women tended to eat chicken soup for more than 15 days (80.3%) and sesame oil chicken for more than 7 days (70.2%). Only 4.7% of the participants did not take any Chinese medicines during the first month after delivery. Most women took Sheng-hau-tang (91.1%) and Si-wu-tang (87.2%) for 1–10 days. The number of days the women took *E. ulmoides* ranged from 0 to more than 21 days.

### 3.3. Uterine Involution Indices 4–6 Weeks after Delivery

The mean AP diameter of the uterus 5 cm from the fundus was 42.4 mm, and that of the cavity was 6.1 mm. The maximum AP diameter of the uterus was 47.2 mm, and that of the cavity was 8.5 mm (Table 4). The cavity was empty in 125 of 127 women. The cervical area was also empty in 125 of 127 women. Gas or hyperechogenic focus was detected in 17 of 127 women, and 46 of 127 women had an anteverted uterus. There were no differences in the indices of uterine involution between women with natural spontaneous delivery and those who underwent Cesarean delivery.

### 3.4. The Relationship between the Use of Dietary and Herbal Therapies and AP Diameter of the Uterus and Cavity

Table 5 shows the relationship between each variable of herbal and dietary therapies and the AP diameter of the uterus and the cavity. Pearson correlation analysis revealed that consumption of sesame oil chicken correlated positively with maximum AP diameter of the uterus (*r* = 0.153, *P* = .043). Total herb taking correlated negatively with maximum AP diameter (*r* = −0.166, *P* = .031) and the AP diameter 5 cm from the fundus (*r* = −0.194, *P* = .014). Taking *E. ulmoides* was negatively correlated with the maximum AP diameter of the uterus (*r* = −0.307, *P* = .001), uterine AP diameter 5 cm from the fundus (*r* = −0.285, *P* = .002), and maximum AP diameter of the cavity (*r* = −0.245, *P* = .006).

Variables in the same category were simultaneously entered into a multivariate linear regression model. The results showed that dishes only accounted for 2.5% of the variance in maximum AP diameter of uterus and eating sesame oil chicken did not show a significant effect in the multivariate analysis (95% CI = −0.131, 2.035). The summative variable, total herb taking, accounted for 2.8% of the variance in maximum AP diameter of uterus and 3.8% of the variance in AP diameter 5 cm from the fundus. After adjusting for other covariates, total herb taking was associated with uterine AP diameter 5 cm from the fundus (*B* = −1.331, 95% CI = −2.521, −0.140) but was not associated with maximum AP diameter of the uterus (95% CI = −2.05, 0.60). The three herbal medicines accounted for 10.9% of the variance in the maximum AP diameter of uterus, 9.1% of the variance in AP diameter 5 cm from the fundus, and only 6.1% of the variance in the maximum AP diameter of the cavity. Taking *E. ulmoides* was negatively correlated with the maximum AP diameter of the uterus (*B* = −2.048, 95% CI = −3.246, −0.851), the AP diameter of the uterus 5 cm from the fundus (*B* = −1.730, 95% CI = −2.825, −0.635), and the AP diameter of the cavity (*B* = −  0.657, 95% CI = −1.212, −0.102).

**Figure 2:**
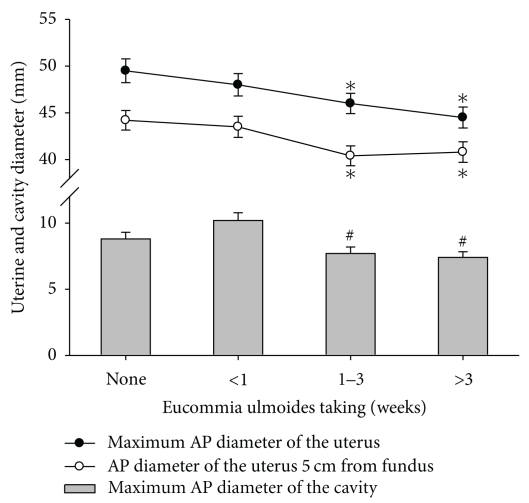
Differences in AP diameter of the uterus and cavity among women who took *E. ulmoides* for zero days, for less than 1 week, for 1–3 weeks, and more than 3 weeks. *P < .05 compared with women who did not take *E. ulmoides*; ^#^ *P* < .05 compared with women who took *E. ulmoides* for less than 1 week.

One-way ANOVA was employed to detect differences in the AP diameter of the uterus and the cavity among women taking *E. ulmoides* for 0 days, less than 1 week, 1 to 3 weeks, and more than 3 weeks (Figure 2). The maximum AP diameter of the uterus and the AP diameter of the uterus 5 cm from the fundus were smaller in women who took *E. ulmoides* for 1 to 3 weeks and in women who took the drug for more than 3 weeks than in women who did not take *E. ulmoides* (*P* < .05). Moreover, the maximum AP diameter of the cavity was smaller in women who took *E. ulmoides* for 1 to 3 weeks and in women who took the drug for more than 3 weeks than in women who took *E. ulmoides* for less than 1 week (*P* < .05).

### 3.5. The Relationship between the Use of Diet and Herb and the Presence of Gas/Hypoechogenic Foci and Anteverted Uterus

In this study, women with empty cavities or cervical areas highly outnumbered those with contents in the cavity or the cervical areas (both 125 versus 2). Therefore, those 2 indices were not considered in the correlation analysis. The presence of gas/hypoechogenic foci inside the uterine cavity and the anteversion of the uterus were recorded as yes or no, and biserial correlation analysis showed that Sheng-hau-tang and Si-wu-tang were positively correlated with the presence of the anteverted uterus (Table 7). Results of logistic regression analysis showed that only Sheng-hau-tang was the predictor of anteverted uterus (*P* = .03; OR = 1.846, 96% CI ranged 1.063–3.206; Table 8).

## 4. Discussion

Traditional Chinese postpartum care is believed to facilitate the uterine recovery for women after delivery [6]. In this study, we investigated the relationship between the uterine involution and the use of dietary therapy and herbal therapy within the first month after delivery. We found that the herbal therapy used in traditional Chinese postpartum care correlated with uterine involution, and that its use was a predictor for uterine involution indices. Among the herbal medicines, the consumption of *E. ulmoides* negatively correlated with the AP diameter of the uterus and the cavity. Moreover, the consumption of Sheng-hau-tang correlated with the presence of anteverted uterus 4 to 6 weeks after delivery.

 In our study, the maximum AP diameter of uterus and the cavity and that measured 5 cm from the fundus were similar to those reported in a previous study that longitudinally recorded the involution process in mothers with normal delivery [2]. We recruited women who underwent Cesarean delivery in addition to mothers with natural spontaneous delivery because the rate of Cesarean delivery in Taiwan is higher than that in many Western countries [22]. We found that there were no differences in the AP diameter of the uterus and cavity, or the presence of an empty cavity, empty cervical area, gas/hypoechogenic foci, and an anteverted uterus between women with natural spontaneous delivery and women who underwent Cesarean delivery. 

 We divided dietary therapy in the Chinese postpartum care into two categories for analysis: food nature and dish. A previous study showed that adherence to traditional Chinese postpartum care, including avoiding “hot” and “cold” food, reduced the severity of physical symptoms related to delivery, such as back pain, joint pain, wound pain, and hemorrhoids [11]. On the other hand, dishes made of chicken, including two of the most popular Taiwanese dishes, sesame oil chicken soup and chicken soup, are believed to enhance nutrient/protein intake and are, therefore, considered beneficial to postpartum women [23]. In our study, none of the three types of extreme food correlated with the uterine involution indices, neither did the consumption of chicken soup. Although there was a borderline significant correlation between the use of sesame oil chicken and maximum uterine AP diameter in Pearson correlation analysis, the significance disappears when analyzed by multivariate linear regression model. The dishes contributed only 2.5% of the variance in maximum AP diameter of the uterus. We proposed that the role of dietary therapy is more in nutritional supplement, than in the uterine involution.

 The bark of *E. ulmoides* (also called Du-Zhong or Tu-Chung) is a herb used to prevent and treat pelvic girdle pain and lower back pain conditions that affect approximately 25% of all women in the postpartum period [24]. *E. ulmoides* has been shown to strengthen tendon and bones, reinforce muscle, prevent miscarriage [25, 26], increase bone mineral density [27], and lower blood pressure [28]. In this study, the negative correlation between* E. ulmoides* and AP diameter of the uterus implies that *E. ulmoides* induces uterine involution by diminishing uterine size. The maximum uterine AP diameter and the AP diameter 5 cm from the uterus were significantly smaller in women who took *E. ulmoides* for 1–3 weeks and those in women who took it for more than 3 weeks than in women who did not take the drug. Besides, the maximum AP diameter of the uterine cavity in women who took *E. ulmoides* for 1–3 weeks and that in women who took more than 3 weeks were smaller than the diameter in women who took it for less than 1 week. Therefore, we suggest that *E. ulmoides*, when taken for more than 1 week, can diminish the uterine size and cavity size. Multivariate linear regression also showed that this correlation was not affected by the method of delivery; moreover, the method of delivery did not correlate with any of the involution indices we measured (data not shown). This implies that *E. ulmoides* is effective both in women with natural spontaneous delivery and Cesarean delivery. Our data also showed that the consumption of total herbs was correlated with maximum AP diameter of the uterus and uterine AP diameter measured 5 cm from the fundus. We speculated that *E. ulmoides* accounted for both correlations because there was a significant correlation between *E. ulmoides* and total herb taking via Pearson correlation analysis (*r* = 0.284, *P* = .002). Moreover, the frequency of *E. ulmoides* consumption was the highest among the three herbal medicines we investigated.

Another herbal medicine that was found to correlate with uterine involution was Sheng-hau-tang, a herbal mixture used by about 87% of postpartum women in Taiwan [29]. Women believe that taking Sheng-hau-tang helps in the discharge of lochia and recovery of the uterus [12]. It has been demonstrated that Taiwanese women who take Sheng-hau-tang after delivery have a better life quality than those who do not, especially in terms of role limitations due to physical and emotional problems [30]. However, its effect on the involution process only has been revealed in animal models, which showed that Sheng-hau-tang increases the contraction of the uterus when cotreated with estrogen [31], as well as increases the myoelectric activity of rabbit uterine smooth muscle [32]. Our study found a correlation between Sheng-hau-tang and the presence of anteverted uterus. More than 95.2% of the uterus is retroverted 24 hours after delivery. During the involution, the uterus rotates about 100 to 180 degrees toward the anteverted position, resulting in an 83% decrease in retroversion eight weeks after delivery [2]. Our results implied Sheng-hau-tang might increase the contractile activity and participate in the returning of the uterus to its anteverted position. 

Our study suggests that Chinese medicines are beneficial for women in the recovery of the uterus. However, it is important to emphasize that the methodological problems in the research design limit the interpretation of our results. Our research was exploratory and was performed by cross-sectional measurement and retrospective questionnaires. Therefore, we cannot ascertain the cause-effect relationship between* E. ulmoides *and AP diameter of the uterine and cavity diameter, or between Sheng-hau-tang and anteverted uterus. Controlled trials are needed to clarify the cause. From the present study, we speculate that *E. ulmoides* and Sheng-hau-tang might promote uterine contraction in different mechanism because they correlated with different involution indices. Animal studies are also needed to find the mechanisms that these herbs act through. Moreover, because Chinese medicines and foods are only part of the whole doing-the-month custom, the role that behaviors and life style play in uterine involution also remains to be clarified.

In conclusion, our study was the first to assess the correlation between elements of traditional Chinese postpartum care and uterine involution. We found that *E. ulmoides* was associated with AP diameter of the uterine and the cavity; moreover, Sheng-hau-tang was associated with the presence of anteverted uterus. Based on this study, we suggested that *E. ulmoides* and Sheng-hau-tang might possess contractile activity for postpartum uterus.

## Figures and Tables

**Table 1:** The ingredients of Sheng-hau-tang and Si-wu-tang.

Sheng-hau-tang (standard dose)
*Angelica sinensis* (24 g)
*Ligusticum chuanxiong* (9 g)
*Prunus persica *(6 g)
*Zingiber officinale* (2 g)
*Glycyrrhiza uralensis* (2 g)

Si-wu-tang
*Angelica sinensis* (9 g)
*Ligusticum chuanxiong* (6 g)
*Rehmannia glutinosa* (12 g)
*Paeonia lactiflora* (9 g)

**Table 2:** Characteristics of the 127 postpartum women.

Characteristics	*n* (%)
Age	
<25	9 (7.1%)
26–30	53 (41.7%)
31–35	50 (39.4%)
>36	15 (11.8%)

Method of delivery	
Normal spontaneous delivery	89 (70.1%)
Cesarean delivery	38 (29.9%)

Parity	
1	86 (67.7%)
2	33 (26.0%)
≥3	8 (6.3%)

Education	
High school or below	30 (23.6%)
College or university	85 (66.9%)
Graduate school or above	12 (9.4%)

Employment status	
Full time	74 (56.9%)
Part time	5 (4.9%)
Not currently working	48 (38.2%)

Place of staying during 1 month postpartum	
Own or relative's house	109 (85.9%)
Postpartum care center	18 (14.2%)

Place of herbal purchase	
Clinic	27 (21.3%)
Pharmacy	80 (63.0%)
Unspecified	20 (15.7%)

Extra medication during 1 month postpartum	
Antibiotics	16 (12.6%)
Uterotonic agents	8 (6.3%)

**Table 3:** Consumption of diets and herbal medicines during the first postpartum month in the 127 women.

Diet/herb taking	Number (%)			
Food nature	None	1–3 days	4–7 days	>7 days
Ice or cold drink	99 (78.0%)	24 (18.9%)	3 (2.4%)	1 (0.8%)
Cold food	72 (56.7%)	35 (27.6%)	15 (11.8%)	5 (3.9%)
Hot food	81 (63.8%)	37 (29.1%)	7 (5.5%)	2 (1.6%)
Sour food	7 (5.5%)	5 (3.9%)	9 (7.1%)	106 (83.5%)

Dish	0–3 days	3–7 days	7–15 days	>15 days
Chicken soup	2 (1.6%)	7 (5.5%)	16 (12.6%)	102 (80.3%)
Sesame oil chicken	17 (13.4%)	21 (16.5%)	46 (36.2%)	43 (33.9%)

Herb	None	1–4 days	5-10 days	>10 days
Sheng-hau-tang	20 (16.1%)	34 (27.4%)	59 (47.6%)	11 (8.9%)
Si-wu-tang	15 (12.8%)	32 (27.4%)	55 (47.0%)	15 (12.8%)

	None	1–7 days	7–21 days	>21 days

*E. ulmoides *	28 (25.7%)	29 (26.6%)	31 (28.4%)	21 (19.3%)

	None	1–5 days	5–15 days	>15 days

Herb total	6 (4.7%)	14 (11.0%)	38 (29.9%)	69 (54.3%)

**Table 4:** Uterine involution indices among the 127 women.

	Uterine AP diameter mean (SD, range)		Cavity AP diameter mean (SD, range)	
	Maximum	5 cm from fundus
NSD	47.0 (5.7, 32.0–64.3)	42.2 (5.3, 27.1–54.8)	8.6 (2.8, 3.2–15.6)	6.2 (2.2, 2.2–11.5)
C/S	47.5 (7.6, 32.0–69.7)	42.8 (7.0, 29.3–57.4)	8.1 (2.8, 4.0–18)	6.0 (1.8, 2.9–12.3)
Total	47.2 (6.3, 32.0–69.7)	42.4 (5.9, 27.1–57.1)	8.5 (2.8, 3.2–18.0)	6.1 (2.1, 2.2–12.3)

	Empty cavity *n* (%)	Empty cervical area *n* (%)	Gas/hypoechogenic foci *n* (%)	Anteverted uterus *n* (%)

NSD	87 (97.8)	87 (97.8)	12 (13.5)	37 (41.6)
C/S	38 (100)	38 (100)	5 (13.2)	9 (23.7)
Total	125 (98.4)	125 (98.4)	17 (13.4)	46 (36.2)

NSD: natural spontaneous delivery; C/S: Cesarean delivery.

**Table 5:** Pearson correlation between the diet/herb consumption and AP diameter of the uterus and cavity.

Diet/herb taking	Uterine		Cavity	
	Maximum	5 cm from fundus	Maximum	5 cm from fundus
Food nature				
Ice or cold drink	0.069	0.095	0.058	0.042
Cold food	0.053	0.027	−0.054	−0.082
Hot food	−0.074	−0.026	0.010	−0.042
Sour food	−0.051	−0.057	0.076	0.050

Dish				
Chicken soup	−0.032	−0.048	−0.027	−0.011
Sesame oil chicken	0.153^a^	0.133	0.119	0.139

Herb				
Sheng-hau-tang	0.045	0.025	−0.076	−0.052
Si-wu-tang	−0.055	−0.010	−0.064	−0.052
*E. ulmoides *	−0.307^d^	−0.285^e^	−0.245^f^	−0.155
Total	−0.166^b^	−0.194^c^	−0.107	−0.067

AP, anteriorposterior; ^a^ *P* = .043; ^b^ *P* = .031; ^c^ *P* = .014; ^d^ *P* = .001; ^e^ *P* = .002; ^f^ *P* = .006.

**Table 6:** Multivariate linear regression model for factors associated AP diameter of the uterus and cavity.

	Uterine AP diameter				Cavity AP diameter			
	Maximum		5 cm from fundus		Maximum		5 cm from fundus	
Diet/herbs	*B* (95% CI)	Beta (*S*)	*B* (95% CI)	Beta (*S*)	*B* (95% CI)	Beta (*S*)	*B* (95% CI)	Beta (*S*)

Food nature								
Ice or cold drink	1.509 (−0.970, 3.989)	0.129	1.474 (−0.853, 3.800)	0.135	0.560 (−0.567, 1.687)	0.106	0.450 (−0.378, 1.278)	0.116
Cold food	0.574 (−0.840, 1.990)	0.077	0.191 (−1.138, 1.519)	0.027	−0.251 (−0.894, 0.392)	−0.074	−0.215 (−0.688, 0.257)	−0.087
Hot food	−1.539 (−3.546, 0.469)	−0.165	−0.887 (−2.771, 0.997)	−0.102	−0.105 (−1.017, 0.808)	−0.025	−0.236 (−0.906, 0.435)	−0.076
Sour food	−0.149 (−1.592, 1.293)	−0.019	−0.207 (−1.561, 1.146)	−0.028	0.342 (−0.314, 0.997)	0.095	0.199 (−0.283, 0.681)	0.076

Dish								
Chicken soup	−0.359 (−2.080, 1.362)	−0.037	−0.447 (−2.089, 1.135)	−0.052	−0.138 (−0.921, 0.644)	−0.031	−0.050 (−0.624, 0.525)	−0.015
Sesame oil chicken	0.952 (−0.131, 2.035)	0.154	0.774 (−0.240, 1.789)	0.134	0.335 (−0.157, 0.827)	0.120	0.287 (−0.074, 0.648)	0.140

Herb								
Sheng-hau-tang	−0.941 (−0.511, 2.392)	0.126	0.646 (−0.682, 1.974)	0.096	−0.058 (−0.731, 0.616)	−0.017	−0.033 (−0.523, 0.485)	−0.014
Si-wu-tang	−0.149 (−1.584, 1.285)	−0.020	0.168 (−1.144, 1.480)	0.025	−0.093 (−0.758, 0.572)	−0.027	−0.069 (−0.554, 0.415)	−0.029
*E. ulmoides *	−2.048* (−3.246, −0.851)	−0.334	−1.730* (−2.825, −0.635)	−0.312	−0.657* (−1.212, −0.102)	−0.237	−0.292 (−0.696, 0.112)	−0.148
Total	−1.221 (−2.501, 0.060)	−0.166	−1.331* (−2.521, −0.140)	−0.194	−0.354 (−0.938, 0.230)	−0.107	−0.163 (−0.594, 0.269)	−0.067

*B*: understandardized coefficient, beta (*S*): standardized coefficient, and *: *P* < .05.

**Table 7:** Biserial correlation between the diet/herbs and gas/hypoechogenic foci in the cavity and anteverted uterus.

Diet/herbs	Gas/hypoechogenic foci	Anteverted
Food nature		
Ice or cold drink	−0.061	−0.029
Cold food	−0.102	0.039
Hot food	−0.056	−0.089
Sore food	−0.019	−0.073

Dish		
Chicken soup	−0.043	0.027
Sesame oil chicken	0.128	0.054

Herb		
Total	0.007	−0.030
Sheng-hau-tang	0.064	0.233^a^
Si-wu-tang	0.123	0.207^b^
*E. ulmoides *	−0.120	0.078

^a^ *P* = .009; ^b^ *P* = .018.

**Table 8:** Odds ratios in logistic regression model for gas/hypoechogenic foci in the cavity and anteverted uterus.

Diet/herbs	Gas/hypoechogenic foci	Anteverted
Food nature		
Ice or cold drink	0.771 (0.206, 2.878)	0.956 (0.416, 2.242)
Cold food	0.698 (0.316, 1.501)	1.225 (0.770, 1.948)
Hot food	0.986 (0.359, 2.705)	0.700 (0.352, 1.392)
Sour food	0.926 (0.497, 1.727)	0.841 (0.531, 1.332)

Dish		
Chicken soup	0.803 (0.382, 1.688)	1.087 (0.608, 1.942)
Sesame oil chicken	1.536 (0.857, 2.755)	1.116 (0.778, 1.602)

Herb		
Total	1.023 (0.559, 1.872)	0.930 (0.610, 1.416)
Sheng-hau-tang	1.447 (0.696, 3.008)	1.846* (1.063, 3.206)
Si-wu-tang	1.681 (0.818, 3.458)	1.724 (0.995, 2.985)
*E. ulmoides *	0.611 (0.330, 1.129)	0.999 (0.653, 1.528)

Data were expressed as odds ratio (95% CI) and *P = .030.
